# Phase Angle and Impedance Ratio as Indicators of Physical Function and Fear of Falling in Older Adult Women: Cross-Sectional Analysis

**DOI:** 10.2196/53975

**Published:** 2024-03-05

**Authors:** Danielle A Sterner, Jeffrey R Stout, Kworweinski Lafontant, Joon-Hyuk Park, David H Fukuda, Ladda Thiamwong

**Affiliations:** 1 Physiology of Work and Exercise Response (POWER) Lab, Institute of Exercise Physiology and Rehabilitation Science, University of Central Florida, Orlando, FL, United States; 2 Disability, Aging, and Technology Cluster, University of Central Florida, Orlando, FL, United States; 3 College of Nursing, University of Central Florida, Orlando, FL, United States; 4 Mechanical and Aerospace Engineering Department, University of Central Florida, Orlando, FL, United States

**Keywords:** handgrip, sit-to-stand, fitness, assessment, functionality, body composition, balance, fall, impedance, bioelectrical

## Abstract

**Background:**

Older adults experience a significant decline in muscle integrity and function with aging. Early detection of decreased muscle quality can pave the way for interventions to mitigate the progression of age-related physical declines. Phase angle (PhA) and impedance ratio (IR) are measures of muscle integrity, which can be assessed quickly via bioelectrical impedance analysis (BIA) and may be indicative of physical function.

**Objective:**

This study aimed to characterize the relationships among handgrip strength (HGS), sit-to-stand (STS), BTrackS balance scores, fear of falling (evaluated using the Short Falls Efficacy Scale–International [Short FES-I]), and IR among community-dwelling older adult women classified as having a low or high PhA.

**Methods:**

A cross-sectional analysis was conducted with 85 older women (mean age 75.0, SD 7.2 years; mean weight 71.0, SD 15.0 kg; mean height 162.6, SD 6.1 cm). To examine the influence of PhA on performance measures, participants were divided into 2 PhA groups: high (>4.1°; n=56) and low (≤4.1°; n=29). Data were nonnormative; hence, the Mann-Whitney *U* test was used to evaluate between-group differences, and Kendall τ coefficients were used to determine the partial correlations.

**Results:**

The low PhA group had a significantly higher IR (mean 0.85, SD 0.03) than the high PhA group (mean 0.81, SD 0.03; *r*=.92; *P*<.001). The high PhA group had superior HGS (mean 21.4, SD 6.2 kg; *P*=.007; *r*=0.36), BTrackS balance scores (mean 26.6, SD 9.5 cm; *P*=.03; *r*=0.30), and STS scores (mean 16.0, SD 5.5; *P*<.001; *r*=0.49) than the low PhA group (mean HGS 17.6, SD 4.7 kg; mean BTrackS balance score 37.1, SD 21.1 cm; mean STS score 10.7, SD 6.2). Both PhA and IR were significantly correlated with HGS and BTrackS balance, STS, and Short FES-I scores (*P*<.05). However, on adjusting for the whole sample’s age, only PhA was strongly correlated with HGS (τb=0.75; *P*=.003) and STS scores (τb=0.76; *P*=.002). Short FES-I scores were moderately correlated with IR (τb=0.46; *P*=.07) after controlling for age. No significant between-group differences were observed for height, weight, or BMI.

**Conclusions:**

PhA and IR are associated with physical function and the fear of falling in older women. However, only PhA was significantly associated with physical function (HGS and STS) independent of age. Conversely, only IR was significantly associated with the fear of falling. Diminished physical function and increased IR appear to be characteristics of older women with a PhA of ≤4.1°. These findings suggest that PhA and IR measured through BIA together may serve as a valuable tool for early identification of older women at the risk of functional decline and a heightened fear of falling.

## Introduction

By 2060, the population of American adults older than 65 years is projected to surge, doubling from 52 million in 2018 to 95 million [1]. Muscle weakness and functional loss contribute to falls; in 2020, approximately 3 million adults older than 65 years were treated in emergency rooms as a result of falling, with 800,000 of them having been hospitalized [2]. The annual cost of falls has exceeded US $50 billion, with Medicare and Medicaid covering 75% of these costs [2]. These older adults often experience a significant decline in muscle quality, quantity, and function [3]. Such age-related functional limitations can make everyday tasks, such as cooking, cleaning, and interacting with grandchildren, increasingly challenging [3]. The natural aging process brings about changes in body composition, often directly affecting physical function, as seen in sarcopenia [4]. Sarcopenia is characterized by a rapid loss of muscle strength with aging and is significantly associated with physical disability, decreased quality of life, and increased mortality [5,6]. Moreover, aging exacerbates traits of frailty, a syndrome characterized by a decrease in reserve capacity across various physiological systems, reducing their ability to withstand minor stressors [7,8]. This can result in an increased risk of falls, fractures, and disabilities [9,10], as well as higher mortality rates [7,8].

Aging also affects the musculoskeletal system, causing a decrease in skeletal muscle integrity, mass, strength, and function, along with an increase in the accumulation of noncontractile and adipose tissue [11,12]. Decreases in skeletal muscle integrity can result from a diminished cross-sectional area of muscle fibers, a transition from type II to type I fibers, and a loss of innervation [13]. A loss of skeletal muscle integrity can manifest through impaired balance and subsequently decreased physical function [13]. While not a physical characteristic, a fear of falling can also impose a restriction on physical activity and exacerbate functional loss [14]. Brouwer et al [15] assessed the fear of falling in healthy older adults and concluded that fear of falling was associated with poorer physical function characterized by a lower walking speed and lower limb weakness. Commonly assessed through questionnaires, early detection of the fear of falling can lead to interventions designed to increase physical activity in older adults and mitigate functional loss [16]. Likewise, early detection of decreased muscle quality can pave the way for interventions that may mitigate the clinical progression of sarcopenia and frailty [17].

Bioelectrical impedance analysis (BIA) is a quick, noninvasive, cost-effective method for assessing body composition within the Two-Compartment Model. It has also gained popularity, especially among older populations, as an assessment for skeletal muscle integrity and cellular health [10]. The BIA involves directing a constant low-level electrical current through the body [18]. This system has 3 main components: reactance (X_c_), which measures the ability of cell membranes to store electrical charge (capacitance); resistance (R), which represents the resistive properties of cells due to intracellular water (ICW) and extracellular water (ECW) [19]; and impedance (Z), which represents the overall opposition to the electrical current [20]. Impedance provides another opportunity to examine cellular integrity with body cell mass [21]. Impedance ratio (IR) measures Z at high and low frequencies (in kHz) and can be indicative of possible cell membrane dysfunction based on body cell mass [22]. The IR for the whole body, upper limbs, and lower limbs is commonly calculated at 250 kHz or 5 kHz [21,23]. At higher frequencies, Z can penetrate cell membranes, therefore allowing for total body water (ECW + ICW) to be measured; however, at lower frequencies, Z can only measure ECW [24]. An IR ratio closer to 1 is indicative of cell membrane disruption, allowing more fluids, proteins, and electrolytes to shift into the extracellular space [22]. A strong inverse correlation has been reported between the phase angle (PhA) and IR in different clinical populations [22]. PhA is defined as the delay in current flow caused by a reduction in cell membrane capacitance [25].

PhA is calculated as the ratio of R to X_c_ at a frequency of 50 kHz, as measured through BIA [26]. Furthermore, PhA is influenced by hydration status and lean body mass [27]. Therefore, it directly relates to the electrical functioning of cell membranes, skeletal muscle integrity, and PhA itself [12,27]. Higher PhA values are indicative of superior cell membrane integrity and cellular health [10]. Disease, dehydration, inflammation, malnutrition, and functional disabilities can cause disturbances in electrical tissue properties, reflected by a lower PhA [28-30]. A low PhA increases the risks for disability, falls, sarcopenia, frailty, and mortality among older populations [29]. PhA can be used as a proactive measure against physical weakening by identifying older individuals at the risk of muscle loss and mortality [10,17,19].

Previous research has established relationships between PhA and handgrip strength (HGS) [31], balance [13], gait speed [32], age [10], sex [30], sarcopenia [33], and BMI [34]. However, previously reported regression models have only been able to account for approximately ≤30% of the variance in PhA [31,35] and have largely left the fear of falling unaccounted for. Therefore, the primary objective of this study was to compare HGS, sit-to-stand (STS) scores, BTrackS balance scores, and fear of falling between groups of older women categorized as having high or low PhA. Additionally, we aimed to assess the relationships among PhA, IR, and physical function metrics while controlling for age, and determine which variables are most strongly associated with PhA and IR in this population. We hypothesized that those with a high PhA would perform better on functional assessments and have a lower fear of falling, and that HGS would have the strongest association with PhA and IR when controlling for age.

## Methods

### Ethical Considerations

All study procedures were conducted in accordance with the tenets of the Declaration of Helsinki, approved by the University of Central Florida’s institutional review board (ID: STUDY00002473), and preregistered on ClinicalTrials.gov (NCT06063187).

### Recruitment

The 85 female participants (n=64, 75% White; n=15, 17% Hispanic; n=4, 5% African American; n=2, 2% Asian) included in this analysis were part of a larger study funded by the National Institutes on Aging (R03AG069799) [36]. This analysis used data from female participants only to specifically characterize the relationships between bioelectrical impedance parameters and physical function metrics in older women. This study used a cross-sectional design to determine whether there were differences in HGS, STS, BTrackS balance scores, fear of falling, and IR based on a low or high PhA among older adult women. The sample of 85 older adult women had a mean age of 75.0 (SD 7.2) years, mean weight of 71.0 (SD 15.0) kg, and mean height of 162.6 (SD 6.1) cm. The study was conducted in low-income communities around central Florida, United States. Recruitment was achieved through flyer distribution, face-to-face engagement, local newsletters, and word of mouth. Community partners and clinical sites facilitated our introduction to potential participants for informed consent, initial screening, and eligibility verification using a checklist. Upon completion of the study, the participants received a US $30 gift card.

Eligible participants met all of the following inclusion criteria: (1) being aged ≥60 years; (2) being of low income status, based on 2019 poverty thresholds relative to family size and the number of children aged ≤18 years [18]; (3) absence of marked cognitive impairment, defined by a memory impairment screen score of ≥5 [37]; and (4) living independently in their own homes or apartments. Exclusion criteria were (1) medical conditions that inhibit balance testing or physical activity, such as the inability to stand on the balance plate or experiencing shortness of breath during physical activity; (2) active treatment from a rehabilitation facility; or (3) the presence of medical implants, such as pacemakers.

### Measurements

#### Grouping of Participants: Low and High PhA

To verify the influence of PhA on performance measures, participants were divided into 2 groups: low PhA (≤4.1°; n=56) and high PhA (>4.1°; n=29). This cutoff was based on previous research that observed a higher prevalence of physical dysfunction and sarcopenia among community-dwelling women aged ≥65 years with a PhA less than 4.1° [34,38].

#### BIA

Body composition was assessed using the InBody s10, a direct segmental multifrequency BIA device from InBody Co, located in Seoul, South Korea. This device is designed to measure Z at 6 different frequencies—1, 5, 50, 250, 500, and 1000 kHz—for both the entire body and individual body segments. All BIA assessments were conducted before performing all other assessments, and all assessments were completed between 9 AM and 12 PM. To ensure accuracy, participants were instructed to fast for a duration of 3-4 hours, abstain from caffeine or alcohol for 24 hours, and avoid exercising for a period of 6-12 hours before testing. Participants were asked to maintain their normal dietary habits and arrive for testing adequately hydrated. On the day of assessment, participants were asked to remove their shoes, socks, and any metallic items. Height and weight were assessed using a digital physician scale and stadiometer (Health-O-Meter, Model 402 KL). Participants were then seated as their skin was prepared with an InBody wipe (InBody Co), and touch-type electrodes were then positioned on their left and right ankles, middle fingers, and thumbs. Participants were required to remain still for 1 minute before the electrodes were removed. PhA was derived by calculating the ratio of R to X_c_ at 50 kHz using the following formula: arc tangent (X_c_/R) × (180/π) [26]. Moreover, IR was determined by dividing Z at 250 kHz by Z at 5 kHz [22,23]. The InBody s10 has good test-retest reliability among adults aged 65 years and older, with an intraclass correlation coefficient (ICC) of 0.82 [39].

#### HGS

Following BIA assessments, HGS was measured using a JAMAR Plus digital handgrip dynamometer (JLW Instruments) to ascertain maximal isometric force in kilograms. Participants, seated with feet flat on the floor and elbow bent at 90°, held the dynamometer in their hand, which was adjusted to allow for a flat second metacarpal and 90° bend at the knuckles. Participants then squeezed the dynamometer as hard as possible for 3-5 seconds across 3 trials, with 30-second rest intervals between each trial. All 3 trials were completed for 1 hand before performing 3 trials with the opposite hand. The maximum value for each hand was recorded, averaged, and used for analysis. The JAMAR handgrip dynamometer is a sound method to test HGS in the clinical setting with good intra- and intertester reliability [36,40].

#### BTrackS Balance Scores

BTrackS balance scores were gauged using the BTrackS balance system (Balance Tracking Systems) following HGS assessments. The BTrackS balance plate and BTrackS Assess Balance software (version 5.5.9) were used to measure center-of-pressure sway during a static stance. The scores and percentile rankings (0-100) were determined in accordance with age group and sex. A score of ≤30 signifies normal balance, while a score of ≥31 indicates poor balance and a moderate to high fall risk [41]. For each trial, participants were instructed to place their hands on their hips, close their eyes, and maintain a static position on the BTrackS Balance Plate for 20 seconds. Participants first underwent a familiarization trial that did not count toward their average score, followed by 3 trials that were averaged into their final score. To mitigate the risk of falls, a piece of sturdy furniture or a walker was placed within the participants’ reach during the test. The BTrackS balance system has been validated and has excellent reliability, with a Pearson correlation coefficient (*r*) of >0.90 and high test-retest reliability with an ICC of 0.83 [41].

#### STS

After BTrackS balance assessments, participants completed the 30-second STS test. STS scores were evaluated by instructing participants to stand up from a chair as many times as possible within 30 seconds. During the test, participants sat in the middle of the chair with their wrists crossed and hands resting on opposite shoulders. The 30-second STS test is a well-validated functional function measure in clinical research and practice, with good test-retest and interrater reliability [42].

#### Short Falls Efficacy Scale–International

Participants were asked to fill out the Short Falls Efficacy Scale–International (Short FES-I) questionnaire, which includes answering 7 questions on a scale of 1-4 that indicates if the participant would be concerned about falling during different activities. In the Short FES-I, a 1 indicates no concern at all and a 4 indicates being very concerned. The Short FES-I has been validated as a predictor of future falls and declines in functional capacity with balance and gait, and it has excellent test-retest ability [16].

### Statistical Analysis

All statistical analyses were conducted using SPSS (version 28; IBM Corp). Descriptive data are presented as mean (SD) values along with ranges where appropriate. The Mann-Whitney *U* test was used to evaluate between-group differences for all variables. A Kolmogorov-Smirnov test determined that the data were nonnormal, so Kendall τ coefficients were used to determine the partial correlations between variables controlled for age. The α value was set considering a *P* value of <.05.

## Results

A total of 88 older women were screened for eligibility and 85 were included in the analysis, with 34% (29/85) of them in the low PhA group and 66% (56/85) of them in the high PhA group. As shown in Table 1, women in the low PhA group were significantly older than those in the high PhA group (*P*=.001). As anticipated, the group with a low PhA had a lower PhA (*P*<.001) and a higher IR (*P*<.001) than those in the high PhA group. In the high PhA group, PhA ranged from 4.2° to 7.0°, and IR ranged from 0.69 to 0.84. In the low PhA group, PhA ranged from 2.5° to 4.1°, and IR ranged from 0.83 to 0.89. No significant between-group differences in height, weight, or BMI were observed. Approximately 52% (44/85) of participants were considered overweight or obese with a BMI of ≥25.9 kg/m^2^.

**Table 1.** Participants’ characteristics and bioelectric impedance analysis (n=85).

Characteristics	Total (n=85), mean (SD)	Low phase angle (n=29), mean (SD)	High phase angle (n=56), mean (SD)	*P* value^a^	Effect size^b^
Age (years)	75.0 (7.2)	79.6 (8.2)	71.0 (5.6)	.001	0.43
Height (cm)	162.6 (6.1)	162.0 (6.0)	163.0 (6.2)	.52	0.09
Weight (kg)	71.0 (15.0)	71.9 (15.7)	71.8 (14.8)	.81	0.03
BMI (kg/m^2^)	26.8 (5.0)	27.3 (5.2)	26.9 (4.9)	.48	0.09
Body fat (%)	33 (10)	38 (9)	31 (10)	.003	0.40
Phase angle (°)	4.4 (0.8)	3.6 (0.4)	4.8 (0.6)	<.001	1.00
Impedance ratio	0.82 (0.03)	0.85 (0.01)	0.81 (0.03)	<.001	0.92

a *P* values refer to the difference between the groups (Mann-Whitney *U* test).

b Effect sizes are provided as rank biserial correlation, whereby 0.10, 0.30, and 0.50 represent small, medium, or large effects, respectively.

Table 2 shows that women in the low PhA group demonstrated poorer physical function than those in the high PhA group. Specifically, the low PhA group had significantly lower average HGS (*P*=.007) and STS scores (*P*<.001). The low PhA group also showed significantly higher balance scores (*P*=.03) and Short FES-I scores (*P*=.001) than the high PhA group.

**Table 2.** Physical function parameters (n=85).

Variable	Low phase angle (n=29), mean (SD)	High phase angle (n=56), mean (SD)	*P* value^a^	Effect size^b^
Average handgrip strength (kg)	17.6 (4.7)	21.4 (6.2)	.007	0.36
Sit-to-stand score	10.7 (6.2)	16.0 (5.5)	<.001	0.49
BTrackS balance score	37.1 (21.1)	26.6 (9.5)	.03	0.30
Short FES-I^c^ score	11.6 (4.2)	9.3 (3.3)	.001	0.42

a *P* values refer to the difference between the groups (Mann-Whitney *U* test).

b Effect sizes are provided as rank biserial correlation, whereby 0.10, 0.30, and 0.50 represent small, medium, or large effects, respectively.

c Short FES-I: Short Falls Efficacy Scale–International.

Kendall rank correlation analysis (Table 3) revealed significant inverse correlations between PhA and age (τb=–0.37; *P*<.001) and between PhA and IR (τb=−0.79; *P*<.001). Significant moderate direct correlations were observed between PhA and STS scores (τb=0.34; *P*<.001) and between PhA and average HGS (τb=0.22; *P*=.002). Small but significant correlations were found between PhA and balance scores (τb=−0.19; *P*=.01) and between PhA and Short FES-I scores (τb=−0.25; *P*=.001). IR had a significant and direct relationship with age (τb=0.37; *P*<.001) and Short FES-I scores (τb=0.26; *P*<.001). IR had a significant and inverse relationship with average HGS (τb=−0.21; *P*=.004) and STS scores (τb=−0.33; *P*<.001).

**Table 3.** Relationships of phase angle and impedance ratio with participant characteristics (n=85).

Variable	Phase angle		Impedance ratio	
	τb^a^	*P* value^b^	τb	*P* value
Age	−0.37	<.001	0.37	<.001
Height	0.09	.25	0.02	.81
Weight	0.06	.39	−0.03	.70
BMI	0.03	.64	−0.04	.60
Impedence ratio	−0.79	<.001	—^c^	—
Average handgrip strength	0.22	.002	−0.21	.004
Sit-to-stand score	0.34	<.001	−0.33	<.001
BTrackS balance score	−0.19	.01	0.18	.02
Short FES-I^d^ score	−0.25	.001	0.26	<.001

a τb=Kendall τ b correlation coefficient.

b *P* values refer to the correlation between variables.

c Not available.

d Short FES-I: Short Falls Efficacy Scale–International.

After controlling for age (Table 4), strong direct correlations were observed between PhA and average HGS (τb=0.75; *P*=.003) and between PhA and STS scores (τb=0.76; *P*=.002). A large direct correlation was observed between IR and Short FES-I scores (τb=0.46; *P*=.07). A moderate inverse correlation was found between IR and STS scores (τb=−0.32; *P*=.20).

**Table 4.** Partial correlations between phase angle and impedance ratio (n=85).

Variable	Phase angle		Impedance ratio	
	τb^a^	*P* value^b^	τb	*P* value
Height	0.50	.04	−0.06	.81
Weight	0.21	.41	0.25	.31
BMI	0.10	.68	0.26	.29
Average handgrip strength	0.75	.003	−0.003	.99
Sit-to-stand score	0.76	.002	−0.32	.20
BTrackS balance score	−0.04	.87	0.11	.66
Short FES-I^c^ score	−0.24	.33	0.46	.07

a τb=Kendall’s τ b partial correlation coefficient.

b *P* values refer to the correlation between variables, controlling for age.

c Short FES-I: Short Falls Efficacy Scale–International.

## Discussion

### Principal Results

The purpose of this study was to examine the relationships among HGS, STS score, balance, fear of falling, PhA, and IR in older adult women classified as having low or high PhA. When comparing physical function between high and low PhA groups, our results demonstrate significant differences in physical function between the high and low PhA groups. We observed a significantly lower IR within the high PhA group (*P*<.001; *r*_rb_=0.92) and a strong negative correlation between PhA and IR (τb=−0.79; *P*<.001). The low PhA group had a significantly higher IR (*P*<.001; *r*_rb_=0.92) and lower PhA (*P*<.001; *r*_rb_=1.0).

When comparing physical function between the high and low PhA groups, our results demonstrate significant differences in physical function between groups. The low PhA group showed significantly lower HGS (*P*=.007; *r*_rb_=0.36), STS scores (*P*<.001; *r*_rb_=0.49), and higher balance scores (*P*=.03; *r*_rb_=0.30) than the high PhA group. Additionally, when examining relationships among PhA, IR, and physical function on controlling for age, moderate correlations were observed between PhA and HGS (τb=0.75; *P*=.003) and STS scores (τb=0.76; *P*=.002). However, balance scores (τb=−0.04; *P*=.87) and fear of falling (τb=−0.24; *P*=.33) showed only weak correlations with PhA when controlling for age. The low PhA group demonstrated a significantly higher Short FES-I score than the high PhA group (*P*=.001; *r*_rb_=0.42).

### Comparison With Previous Literature

Previous literature has demonstrated an increased prevalence of physical dysfunction corresponding with a PhA less than 4.1° in older community-dwelling women, which informed our cutoff value of 4.1° to classify participants as having a low or high PhA [34,38]. Beyond a difference in PhA, the low PhA group also had a significantly lower IR of 0.85 (SD 0.01). This supports previous literature indicating that an IR closer to 1 is indicative of poor cellular health [22]. We observed a significantly lower IR in the high PhA group (Table 1) and a strong negative correlation between PhA and IR (Table 3). This aligns with previous evidence associating both a higher PhA and a lower IR with improved cellular integrity and health [22].

Reduced muscular strength and physical function in older adults has been associated with a lower PhA and higher IR [12,30]. In our study, we did not observe a strong correlation between HGS and PhA (Table 3). This is contrary to previous research, where IR and HGS were significantly correlated (*r*=0.46; *P*<.001) when controlling for age [24]. This discrepancy may be due to differences in methodology; Ballarin et al [24] assessed IR among 19-35–year-olds using a 300 kHz/5 kHz frequency ratio, while our study used a 250 kHz/5 kHz frequency ratio and included participants no younger than 60 years. IR is understood to be lower in younger populations [27], and younger populations still experience increases in HGS. This is contrasted by the higher IR and declining HGS experienced by older individuals.

Few studies have examined the relationship between STS score and PhA. Previous studies have instead used the gait speed test to assess physical function [43]. While STS and gait speed tests are not synonymous, both are dynamic multijoint movements that require both muscular strength and balance. This may explain why STS and gait speed performance are consistently observed as strong predictors of PhA in healthy individuals. This aligns with previous studies showing associations between lower limb strength and PhA [44]. Retaining muscle mass and physical function in the lower legs would have a direct impact on the R, X_c_, and Z measured by BIA via an increase in muscular tissue and intracellular hydration [45]. A recent systematic review and meta-analysis of randomized controlled trials supports this theory, reporting that resistance training of at least 8 weeks increases PhA in older adults [45]. Furthermore, 6 out of the 7 studies included only involved female participants, which aligns with our sample [45]. Within clinical settings where the risks of conducting an STS test or other physical assessments may outweigh the benefits, BIA may serve as a proxy for skeletal muscle quality and physical functioning. PhA has been shown to change with physical functioning longitudinally, as numerous studies have focused on sarcopenia and frailty regarding PhA [29].

The poorer physical function seen in the low PhA group suggests that a lower PhA reflects diminished skeletal muscle integrity and functionality in older adult women. Balance scores were significantly higher in the low PhA group, which are representative of poor balance and moderate to high fall risk (Table 1). A longitudinal study conducted by Asano et al [13] concluded that lower body strength diminishes with aging, and the observed poor balance score was associated with low PhA. We observed a greater fear of falling in the low PhA group (Table 2), and a moderate correlation between IR and fear of falling (Table 4). One study concluded that in women with osteoporosis, slower walking speed, decreased muscular strength, and greater postural sway were correlated with an increased fear of falling [46]. An increase in the fear of falling is associated with decreased muscular strength, which aligns with our findings [15].

### Strengths and Limitations

One of the strengths of this study was the diverse population, including low-income female participants from 4 different racial and ethnic backgrounds. This study also intentionally used portable, accessible, and valid instruments to increase the applicability of the results to clinical practice. However, there are limitations to the study that should be considered. The main findings were raw BIA variables that are directly influenced by fluid distribution throughout the body, as the different frequencies used in calculated IR allow for the assessment of ECW and ICW. Hydration status may thus be a confounding variable as it was not assessed or controlled, although all testing occurred in the late morning for all participants after they were encouraged to void their bladders. In addition, the nonnormal data distribution was accounted for by using robust nonparametric tests during statistical analysis.

### Implications and Future Directions

BIA is a brisk assessment that can be used in older adults to evaluate body composition and cellular health. This study concluded that PhA and IR are both linked to physical function and fear of falling in older women but associate differently when controlling for age. Although they are both measures of cellular health, our study demonstrates how PhA and IR differ in their relationship with physical function and fear of falling. As BIA continues to grow as a clinical assessment, there is a need to better understand how its measures relate to other assessments. A higher IR closer to 1 is indicative of poorer cellular health, which was observed in our low PhA group [22], and is also associated with diminished physical function and a heightened fear of falling in this study. Our study aligns with previous research reporting a strong inverse correlation between PhA and IR [21]; yet, there has been a lack of research investigating IR and its relationship with physical function. IR is a direct reflection of hydration status; therefore, we believe that IR is a helpful passive assessment to use, given the noninvasive and simple nature of the tool. More research on IR in older adults is needed to further examine its relationships with functional assessments.

Aging is associated with a change in body composition and decline in physical function. Therefore, PhA may reflect skeletal muscle health and can be assessed along with physical function. Based on this study, a PhA of ≤4.1° would be indicative of decreased physical function in older women, and an intervention can be implemented to help improve PhA. Additionally, in instances where physical function assessments cannot take place, measuring PhA and IR may be valuable as indicators of physical function for that period. Our results suggest that muscular strength assessments such as HGS and STS may be more closely related to PhA than balance in older adult women. STS scores are indicative of lower body strength, suggesting that lower body strength may be an important factor in PhA and IR. Balance scores can be indicative of muscle integrity, which is associated with frailty, sarcopenia, and malnutrition [29]. It is plausible that lower body strength may be particularly important when evaluating cellular health and physical function via bioimpedance parameters in older women, but more research is needed.

While physical function assessments are commonplace among older adults, psychological and physiological assessments should also be considered. Our results show that the fear of falling has an inverse relationship with physical function and PhA and a direct relationship with IR. Future research should further examine the relationships among fear of falling, physical function, PhA, and IR as they change over time. Understanding how these variables influence each other may aid in designing interventions to improve the health and quality of life of older adults.

### Conclusions

Our results indicate that low PhA (≤4.1°) and high IR are linked with poorer physical function in older women, particularly for HGS and STS ability. PhA and IR are variables that can be assessed regularly during routine checkups and provide an indication of physical function and cellular health. Despite being indicative of cellular integrity and health, IR has not been widely studied in older adults. Assessing hydration status along with BIA measurements may help strengthen the design of future studies. Future research should also compare IR and physical function to our results and assess changes in IR longitudinally within older adults.

## Abbreviations

BIA: bioelectrical impedance analysis
ECW: extracellular water
HGS: handgrip strength
ICC: intraclass correlation coefficient
ICW: intracellular water
IR: impedance ratio
PhA: phase angle
R: resistance
Short FES-I: Short Falls Efficacy Scale–International
STS: sit-to-stand
X_c_: reactance
Z: impedance
